# Evolved Aztreonam Resistance Is Multifactorial and Can Produce Hypervirulence in *Pseudomonas aeruginosa*

**DOI:** 10.1128/mBio.00517-17

**Published:** 2017-10-31

**Authors:** Peter Jorth, Kathryn McLean, Anina Ratjen, Patrick R. Secor, Gilbert E. Bautista, Sumedha Ravishankar, Amir Rezayat, Jayanthi Garudathri, Joe J. Harrison, Rachel A. Harwood, Kelsi Penewit, Adam Waalkes, Pradeep K. Singh, Stephen J. Salipante

**Affiliations:** a Department of Microbiology, University of Washington School of Medicine, Seattle, Washington, USA; b Department of Laboratory Medicine, University of Washington School of Medicine, Seattle, Washington, USA; c Department of Biological Sciences, University of Calgary, Calgary, Alberta, Canada; Emory University School of Medicine

**Keywords:** *Pseudomonas aeruginosa*, antibiotic resistance, aztreonam, cystic fibrosis, fitness, selection, virulence, whole-genome sequencing

## Abstract

While much attention has been focused on acquired antibiotic resistance genes, chromosomal mutations may be most important in chronic infections where isolated, persistently infecting lineages experience repeated antibiotic exposure. Here, we used experimental evolution and whole-genome sequencing to investigate chromosomally encoded mutations causing aztreonam resistance in *Pseudomonas aeruginosa* and characterized the secondary consequences of resistance development. We identified 19 recurrently mutated genes associated with aztreonam resistance. The most frequently observed mutations affected negative transcriptional regulators of the *mexAB-oprM* efflux system and the target of aztreonam, *ftsI*. While individual mutations conferred modest resistance gains, high-level resistance (1,024 µg/ml) was achieved through the accumulation of multiple variants. Despite being largely stable when strains were passaged in the absence of antibiotics, aztreonam resistance was associated with decreased *in vitro* growth rates, indicating an associated fitness cost. In some instances, evolved aztreonam-resistant strains exhibited increased resistance to structurally unrelated antipseudomonal antibiotics. Surprisingly, strains carrying evolved mutations which affected negative regulators of *mexAB-oprM* (*mexR* and *nalD*) demonstrated enhanced virulence in a murine pneumonia infection model. Mutations in these genes, and other genes that we associated with aztreonam resistance, were common in *P. aeruginosa* isolates from chronically infected patients with cystic fibrosis. These findings illuminate mechanisms of *P. aeruginosa* aztreonam resistance and raise the possibility that antibiotic treatment could inadvertently select for hypervirulence phenotypes.

## IMPORTANCE

Inhaled aztreonam is a relatively new antibiotic which is being increasingly used to treat cystic fibrosis patients with *Pseudomonas aeruginosa* airway infections. As for all antimicrobial agents, bacteria can evolve resistance that decreases the effectiveness of the drug; however, the mechanisms and consequences of aztreonam resistance are incompletely understood. Here, using experimental evolution, we have cataloged spontaneous mutations conferring aztreonam resistance and have explored their effects. We found that a diverse collection of genes contributes to aztreonam resistance, each with a small but cumulative effect. Surprisingly, we found that selection for aztreonam resistance mutations could confer increased resistance to other antibiotics and promote hypervirulence in a mouse infection model. Our study reveals inherent mechanisms of aztreonam resistance and indicates that aztreonam exposure can have unintended secondary effects.

## INTRODUCTION

*Pseudomonas aeruginosa* airway infections in cystic fibrosis (CF) are among the most consequential and difficult-to-eradicate chronic infections encountered clinically (1). Infecting *P. aeruginosa* strains commonly evolve antibiotic resistance in response to aggressive long-term antimicrobial treatment (2), and resistance is associated with worse clinical outcomes (3). The *P. aeruginosa* strains that infect people with CF are usually acquired from the environment, and once infection is established, the same *P. aeruginosa* lineage typically persists in a patient’s lungs for decades or more (4–6). There are therefore likely limited opportunities for resident lineages to acquire exogenous antibiotic resistance genes from other *P. aeruginosa* strains via horizontal gene transfer, and consequently, antibiotic resistance in CF frequently arises as a result of spontaneous chromosomal mutations (7, 8). However, the specific mutations underlying resistance phenotypes often remain unknown (2, 9).

Theory predicts that chromosomal mutations which confer antibiotic resistance can reduce bacterial fitness in the absence of antibiotic selection. This is because mutations that optimize one function of a protein commonly reduce its other functions (10–12). For example, specific variants in *Escherichia coli rpsL* confer resistance to streptomycin but compromise the function of the ribosome (13). Consequently, *in vitro* studies have shown that resistance can lead to lower growth rates or other defects compared to wild-type, antibiotic-sensitive strains (13–16).

Nevertheless, other findings have challenged the longstanding theory that antibiotic resistance mutations inherently lead to fitness costs. Classical measures of fitness do not necessarily correlate with the ability of a pathogen to successfully colonize and invade a host, and mutations associated with adverse fitness phenotypes in one environment might not necessarily incur the same costs in another (17). For example, variants in *Mycobacterium tuberculosis* that confer resistance to isoniazid and rifampin affect neither *in vivo* fitness nor competition against wild-type sensitive siblings (18, 19). A transposon (Tn) mutation conferring carbapenem resistance in *P. aeruginosa* actually increases *in vivo* fitness and, surprisingly, virulence (20, 21). Thus, it is possible that some mutations associated with antibiotic resistance have neutral or even positive effects on bacterial performance *in vivo*.

The goals of this study were 2-fold. First, we sought to identify the chromosomal mutations underlying aztreonam resistance *in P. aeruginosa*. Aztreonam is a fully synthetic beta-lactam antibiotic which was approved in an inhaled formulation for chronic suppression therapy in CF patients in 2010 (22). It is now prescribed for nearly half of CF patients infected with *P. aeruginosa* (23), typically for 1-month intervals alternating either with 1 month without treatment or with a month of inhaled tobramycin (24). Multiple clinical trials have reported that *P. aeruginosa* isolates can evolve modest, transient increases in aztreonam resistance during treatment (25–27): the rapid onset of this phenotype suggests that it results from chromosomal mutations. However, only a few mechanisms of aztreonam resistance mediated by spontaneous mutation have been identified (28, 29). The full spectrum of mutations possible and the level of resistance that they confer remain unknown. Second, we sought to understand how aztreonam resistance mutations affected *P. aeruginosa* growth *in vitro* and pathogenesis *in vivo*. To address these questions, we used two strategies of experimental evolution to select for aztreonam resistance in *P. aeruginosa* laboratory strains and performed whole-genome sequencing to identify recurrently mutated genes underlying resistance. We explored the functional consequences of select resistance mutations by characterizing their effects on antimicrobial susceptibility, growth rate, and virulence in murine lung infections.

(Parts of this work were conducted as a thesis project for K. McLean’s master’s dissertation.)

## RESULTS

### Experimental evolution selects for aztreonam-resistant *P. aeruginosa*.

We employed two complementary experimental evolution procedures to artificially select *P. aeruginosa* for aztreonam resistance.

First, we selected three laboratory strains under continuous aztreonam exposure. The reference strain PAO1 was chosen for these experiments because it phylogenomically resembles the *P. aeruginosa* lineages which infect approximately 80% of CF patients (30, 31). We also included related strain MPAO1 because, unlike PAO1, it expresses the *mexEF-oprN* efflux system (32, 33), which could theoretically affect aztreonam resistance (29). Additionally, we selected strain PA14, which resembles the lineages that infect roughly 17% of CF patients (30).

**FIG 1:**
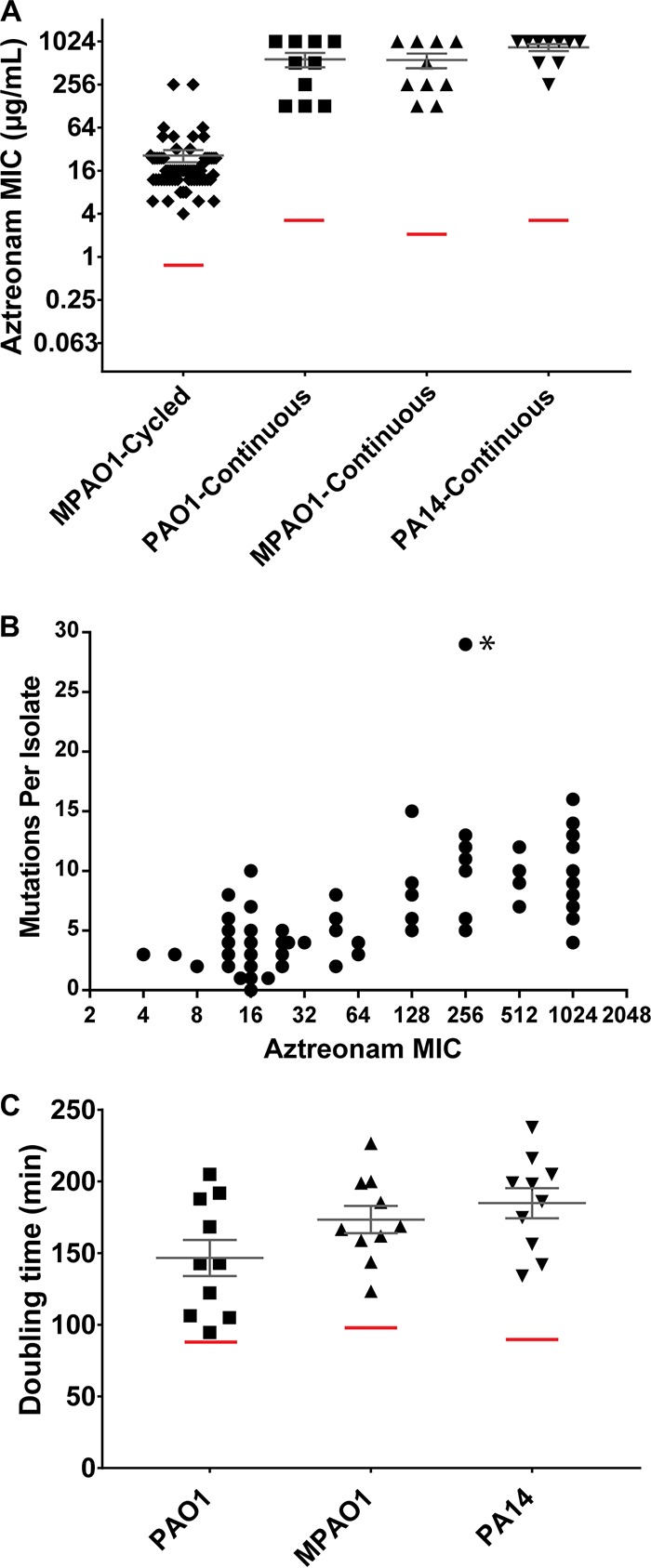
Aztreonam resistance and doubling time of aztreonam-selected strains. (A) Aztreonam MICs of isolates resulting from cyclic and continuous aztreonam selection. Individual isolates are represented by points, with median and standard error of the mean of each group indicated in gray. Control strain MIC is denoted by red lines. (B) Correlation of aztreonam MIC and total mutational burden. Strain G5 (marked by asterisk) was identified as a statistical outlier. (C) Doubling time of evolved strains from continuous selection.

We passaged 10 replicates of each parental strain in the presence of aztreonam and one control replicate without selection. Overnight cultures were initially grown in the absence of antibiotic selection before aliquots from each parent strain were subcultured into fresh medium spanning a range of aztreonam concentrations. After overnight incubation, cells from the highest concentration of antibiotic that supported growth were inoculated into freshly prepared aztreonam-containing medium. We repeated passaging until strains had either achieved growth at 1,024 µg/ml aztreonam or failed to increase in MIC after seven consecutive daily passages. Replicates were then streaked onto aztreonam-containing agar to isolate single colonies for MIC verification and sequencing. The final MICs for passaged replicates (Fig. 1A; see also Table S1 in the supplemental material) were 576 ± 389 µg/ml for PAO1 (average ± standard deviation), 563 ± 389 µg/ml for MPAO1, and 845 ± 282 µg/ml for PA14, representing, on average, a 150- to 200-fold increase from their respective parent strains (4 µg/ml for PAO1, 2 µg/ml for MPAO1, and 4 µg/ml for PA14).

10.1128/mBio.00517-17.4 TABLE S1  Summary of strains used. Download TABLE S1, PDF file, 0.03 MB. Copyright © 2017 Jorth et al. 2017 Jorth et al. This content is distributed under the terms of the Creative Commons Attribution 4.0 International license.

Second, we performed selection to reflect cyclic aztreonam administration used to treat CF patients (24). An overnight culture of MPAO1 grown in the absence of aztreonam was arrayed as 95 replicates onto agar plates spanning a range of aztreonam concentrations (0 to 512 μg/ml). After incubation, a single colony was harvested for each replicate from the highest aztreonam concentration permitting growth and transferred to liquid medium lacking antibiotic for expansion, and selection was then repeated. Twenty-five replicates failed to sustain growth on aztreonam medium over the course of the experiment, resulting in a final count of 70 independently evolved lineages. After nine passages, single colonies from each replicate were isolated. The average MIC of evolved isolates was 27 ± 41 µg/ml (range, 2 to >256 µg/ml), compared to 1 µg/ml for the parental strain (Fig. 1A; Table S1). Notably, these values were on average 2-fold lower than the MICs of strains evolved under continuous aztreonam selection (48 ± 16 µg/ml) after the same number of passages.

### Multiple genes are recurrently mutated in strains passaged in aztreonam.

We performed whole-genome sequencing of the replicates from each passaging experiment to identify chromosomal mutations associated with increased aztreonam resistance. The total mutational burden (number of coding and noncoding changes) for evolved strains compared to their respective parental strains was relatively low: cycled isolates carried an average of 3.8 ± 3.7 (range of 1 to 29) mutations, while continuously selected replicates had higher numbers (PAO1, mean 6.3 ± 1.4 and range of 4 to 9; MPAO1, mean 11.1 ± 2.0 and range of 8 to 15; PA14, mean 12 ± 1.9 and range of 9 to 16 [Table S1]). We found a strong positive correlation between MIC and the number of mutations per strain (Fig. 1B; Pearson’s correlation *r* = 0.675, *P* < 0.0001, excluding strain G5 as a statistical outlier), suggesting that the evolved mutations have a cumulative effect on aztreonam resistance.

Gene mutations which are repeatedly observed after independent exposures to a condition provide strong evidence for adaptive evolution (34). To identify genes that are associated with aztreonam resistance, we therefore focused on genes which were recurrently mutated in at least 20% of replicates derived from a parental strain in each passaging experiment. A total of 19 candidate genes were identified (Table 1). Four recurrently mutated genes were consistently recovered across both passaging experiments and across each of the three strains used: *ftsI*, the gene encoding penicillin binding protein 3 (PBP3), which is the primary target of aztreonam (35), and *mexR*, *nalD*, and *nalC*, which are negative transcriptional regulators of the *mexAB-oprM* efflux system (36). Fifteen additional genes were identified in a smaller subset of strains and had diverse functional roles in efflux system regulation, peptidoglycan biosynthesis and metabolism, amino acid metabolism, energy metabolism, two-component sensor systems, beta-lactamase regulation, and protease activity.

**TABLE 1:** Fraction of isolates with recurrent gene mutations associated with aztreonam resistance

Gene name	Gene function	Fraction of isolates by experimental group:				Fraction of all isolates (*n* = 100)
		Continuous selection			Cycled selection, MPAO1 (*n* = 70)	
		PAO1 (*n* = 10)	MPAO1 (*n* = 10)	PA14 (*n* = 10)		
*mexR*	Efflux regulation	0.80	0.60	0.50	0.35	0.44
*nalD*	Efflux regulation	0.40	0.20	0.30	0.42	0.39
*ftsI*	Penicillin binding protein	0.90	0.70	0.80	0.06	0.28
*phoQ*	Two-component sensor	0.70	0.90	0.80	0.00	0.24
*mexF*	Efflux component	0.00	0.00	0.00	0.27	0.19
*aroB*	Amino acid metabolism	0.90	0.40	0.00	0.00	0.13
*mpl*	Peptidoglycan metabolism	0.00	0.10	0.60	0.08	0.13
*nalC*	Efflux regulation	0.20	0.30	0.40	0.06	0.13
*clpA*	Protease activity	0.40	0.40	0.40	0.00	0.12
*mexT*	Efflux regulation	0.00	1.00	0.00	0.00	0.10
*orfN*	Flagellin glycosylation	0.00	0.00	0.90	0.00	0.09
*pgi*	Energy metabolism	0.10	0.10	0.30	0.00	0.05
*clpS*	Protease activity	0.00	0.00	0.40	0.00	0.04
*PA3206*	Two-component sensor system	0.00	0.20	0.00	0.03	0.04
*dacB*	Penicillin binding protein	0.10	0.10	0.20	0.00	0.04
*pepA*	Protease activity	0.20	0.00	0.20	0.00	0.04
*ampC*	Beta-lactamase precursor	0.00	0.30	0.00	0.00	0.03
*atpA*	Energy metabolism	0.00	0.00	0.20	0.00	0.02
*atpD*	Energy metabolism	0.00	0.00	0.20	0.00	0.02

All strains with high-level aztreonam resistance carried mutations in multiple candidate genes. To explore the temporal staging of mutations during selection, we retrospectively sequenced isolates from each lineage of the continuous antibiotic selection at the time that they first achieved clinical levels of aztreonam resistance (32 µg/ml) and compared the mutations that they carried to those present in lineage-matched isolates from the conclusion of passaging (Table S2). Mutations in *ftsI*, *mexR*, *nalD*, *nalC*, and *phoQ* were present in 28 of 30 isolates from the earlier time point as well as in the terminally evolved isolates. Most other recurrent mutations were exclusively identified in isolates from the endpoint of selection, including genes involved in peptidoglycan recycling (*mpl* and *dacB*), energy metabolism (*atpA*, *atpD*, and *pgi*), beta-lactamase production (*ampC*), and protease production (*clpS*). These findings suggest that mutations in some genes are preferentially selected during the evolution of high-level resistance and that these resistance mutations are either not beneficial or not tolerated in the absence of early-occurring variants.

10.1128/mBio.00517-17.5 TABLE S2  Temporal staging of aztreonam resistance mutations. Download TABLE S2, PDF file, 0.02 MB. Copyright © 2017 Jorth et al. 2017 Jorth et al. This content is distributed under the terms of the Creative Commons Attribution 4.0 International license.

### Inactivating recurrently mutated genes increases aztreonam resistance.

Many of the candidate resistance genes identified in passaged strains carried nonsense and frameshift mutations, indicating that gene-inactivating mutations were often selected during aztreonam passaging. To verify that disruption of candidate genes could lead to increased aztreonam resistance, we tested the effects of inactivating some candidate genes using transposon mutants (37, 38).

We performed aztreonam susceptibility testing on the 22 MPAO1 and 8 PA14 transposon mutants which were available for 16 of our genes of interest (Table S3). Most transposon mutants (18 of 30) exhibited 2- to 8-fold increases in aztreonam resistance compared to both wild-type parent strains (MPAO1 and PA14), while a phenotypically neutral MPAO1 transposon mutant control had no changes in susceptibility for 11 different antibiotics (assessed using Biolog phenotype microarray). Although *phoQ*, *pepA*, and *mpl* transposon mutants exhibited an average of approximately 2-fold higher MICs than that of wild-type MPAO1, orthologous PA14 transposon mutants had less robust increases than those of their own parental strain. Considering that PA14 had a higher baseline aztreonam MIC than did MPAO1, this result could reflect strain-specific differences. Only the MPAO1 *aroB* transposon mutant did not confer increased resistance to aztreonam. However, most mutations identified in *aroB* (as well as *phoQ*, *pepA*, and *mpl*) were missense ones, and therefore, insertional disruption of these genes by transposon mutagenesis may not model more subtly disruptive or gain-of-function effects for these genes.

10.1128/mBio.00517-17.6 TABLE S3  MIC results of transposon mutants. Download TABLE S3, PDF file, 0.02 MB. Copyright © 2017 Jorth et al. 2017 Jorth et al. This content is distributed under the terms of the Creative Commons Attribution 4.0 International license.

### Constant antibiotic exposure selects for resistant strains with growth defects.

The acquisition of mutations associated with antibiotic resistance can produce variable fitness costs, including a reduced growth rate in the absence of antibiotics (39). To determine whether aztreonam resistance led to generalized defects in growth, we measured generation times of highly resistant isolates from the continuous selection experiments when strains were grown in rich medium. Compared to their matched parent strains, 29 of 30 evolved lineages had significantly lower growth rates when cultured in rich broth (Student’s two-tailed *t* test, *P* ≤ 0.0023) (Fig. 1C; Table S4). However, among resistant isolates, generation times did not correlate with aztreonam MIC (Pearson’s correlation *r* = 0.1734, *P* = 0.3595), indicating no relationship between resistance level and the degree of slowed growth.

10.1128/mBio.00517-17.7 TABLE S4  Doubling time analyses. Download TABLE S4, PDF file, 0.03 MB. Copyright © 2017 Jorth et al. 2017 Jorth et al. This content is distributed under the terms of the Creative Commons Attribution 4.0 International license.

### Aztreonam resistance is largely stable in the absence of antibiotic selection.

The growth defects associated with aztreonam resistance suggest a fitness cost which could be unfavorable in the absence of antibiotic selection. To explore this possibility, we assessed the stability of aztreonam resistance in the absence of antibiotic selection. We passaged isolates with high-level resistance from the continuous selection experiment daily in rich medium in the absence of drug for 4 weeks, with weekly evaluation of MICs (Table S5). Aztreonam resistance levels were unaltered by passaging in 21 of 30 lineages (70%). Although 9 isolates did exhibit a measurable decrease in MIC, all maintained MICs that were more than 9-fold greater than that of the passaged parent strain (32 to 256 µg/ml).

10.1128/mBio.00517-17.8 TABLE S5  MIC of isolates passaged without selection. Download TABLE S5, PDF file, 0.02 MB. Copyright © 2017 Jorth et al. 2017 Jorth et al. This content is distributed under the terms of the Creative Commons Attribution 4.0 International license.

We performed whole-genome sequencing of isolates which experienced a reduction in MIC during passaging without aztreonam to explore the genomic changes associated with loss of aztreonam resistance (Table S6). Two isolates carried reversions in recurrently mutated resistance genes (*aroB* in strain HP6 and *mpl* in strain H43). Two isolates (strains H41 and H43) retained the polymorphisms carried by the high-resistance parent and accumulated one to six additional mutations in genes not identified as being associated with aztreonam resistance in our study or the primary literature. Two isolates (strain HM8 and strain H42) had reversion mutations in genes not recurrently identified across strains in this study and in evolutionarily conserved noncoding intragenic regions. Surprisingly, despite ample sequence coverage (Table S7) the two remaining isolates (strains HM2 and HM7), which had 8-fold and 4-fold decreases, respectively, in MIC after the first week of passaging, carried no identifiable sequence changes. This result potentially suggests epigenetic or regulatory changes in these strains.

10.1128/mBio.00517-17.9 TABLE S6  Sequence variants identified in isolates passaged without selection. Download TABLE S6, PDF file, 0.04 MB. Copyright © 2017 Jorth et al. 2017 Jorth et al. This content is distributed under the terms of the Creative Commons Attribution 4.0 International license.

10.1128/mBio.00517-17.10 TABLE S7  Summary of sequence read coverage per isolate. Download TABLE S7, PDF file, 0.02 MB. Copyright © 2017 Jorth et al. 2017 Jorth et al. This content is distributed under the terms of the Creative Commons Attribution 4.0 International license.

We next determined whether loss of the aztreonam resistance phenotype also reduced generation times. Surprisingly, no consistent trend was observed between growth rates and MIC after passaging in the absence of aztreonam selection (Table S4). Five of the nine strains with reduced aztreonam resistance had statistically significant (*P* ≤ 0.0217, Student’s two-tailed *t* test) reductions in doubling time compared to their unpassaged progenitors. Surprisingly, 16 of the 21 passaged strains which retained their original levels of aztreonam resistance also showed significantly more rapid growth. These findings suggest that loss of the aztreonam resistance phenotype is not necessarily driven by selection for corrected growth defects.

### Aztreonam selection can lead to multidrug resistance.

**FIG 2:**
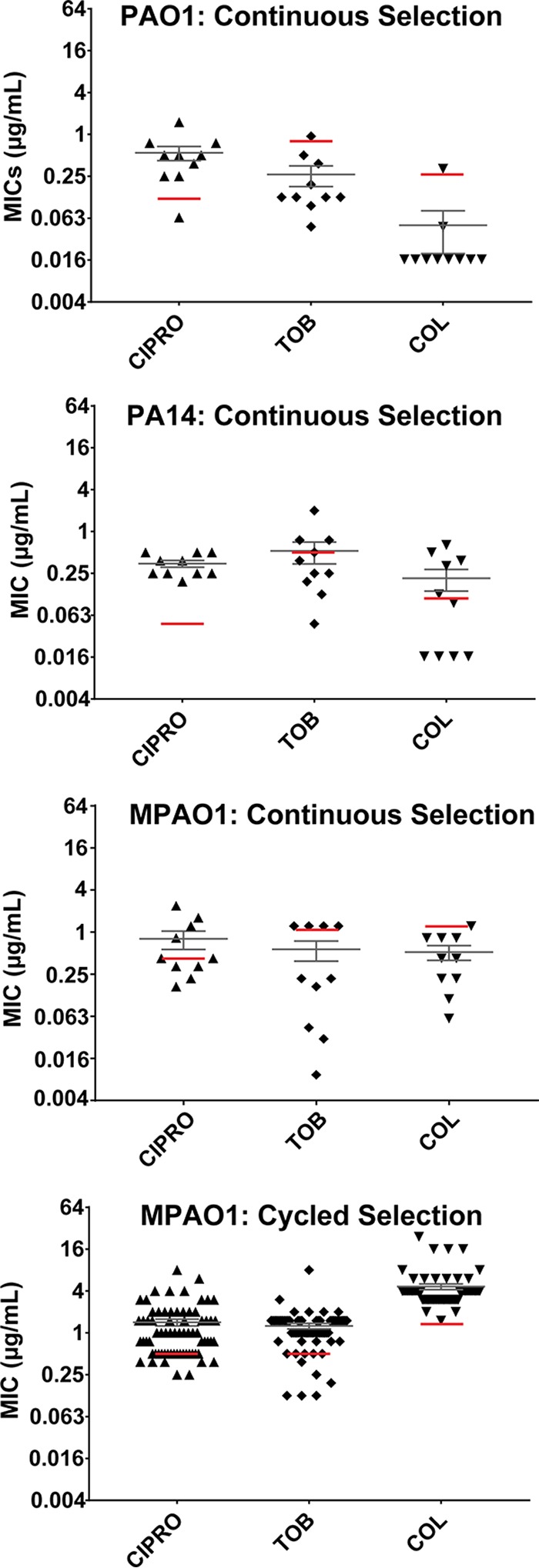
Multidrug susceptibility testing of aztreonam-resistant isolates. MICs of isolates resulting from cyclic and continuous aztreonam selection on three different antibiotics (ciprofloxacin, tobramycin, and colistin) used for CF airway infections. Individual isolates are represented by points, with median and standard error of the mean of each shown by gray bars. Control strain MIC is denoted by red lines.

Several recurrent mutations from our *in vitro* evolution experiments affected antibiotic efflux genes, which can promote resistance to multiple antibiotics (36, 40–42). We therefore tested the hypothesis that strains selected for aztreonam resistance could display increased resistance to multiple drugs. Passaged strains were subjected to antimicrobial susceptibility testing with tobramycin, colistin, and ciprofloxacin (Fig. 2), which are antipseudomonal antibiotics used to treat CF patients that target cellular processes unrelated to those affected by aztreonam.

Aztreonam-passaged isolates from the cyclic evolution experiment on average had higher MICs for all three antibiotics than did the parental MPAO1 strain. Greater than 80% of cycled replicates were ≥2-fold more resistant to at least one antibiotic in addition to aztreonam. While 60 individual isolates had equal or greater levels of resistance to tobramycin, colistin, and ciprofloxacin relative to the parent, 10 isolates from this selection scheme proved more susceptible to one or more agents. In contrast, continuously passaged MPAO1 isolates exhibited decreased resistance to tobramycin and colistin relative to the parental strain and an average 2-fold increase in ciprofloxacin resistance. Continuously passaged PAO1 and PA14 also had decreased resistance to tobramycin and colistin and, on average, a 5-fold increase in ciprofloxacin resistance. There was a significant (*P* = 5 × 10^−5^, two-tailed Student’s *t* test) association between mutation of *mexAB-oprM* regulators and increased ciprofloxacin resistance but no significant correlation between these mutations and increased resistance to other antibiotics.

### *P. aeruginosa* strains passaged in aztreonam are hypervirulent.

The most common and earliest-occurring aztreonam resistance mutations across all selection experiments affected transcriptional regulators of the *mexAB-oprM* efflux pump. Most evolved strains carried disruptive mutations in *nalD* (39 of 100) or *mexR* (44 of 100), which are predicted to result in *mexAB-orpM* overexpression (36, 43). It has previously been reported that inactivation of *mexA* leads to decreased virulence in a murine infection model (21), leading us to hypothesize that inactivating mutations in *mexAB-oprM* negative regulators could simultaneously increase aztreonam resistance and virulence.

To test this hypothesis, two evolved strains from the cyclic passaging experiment carrying nonsynonymous mutations in *nalD* (T158P) or *mexR* (E118*) were selected. To initially determine whether the *nalD* and *mexR* mutations were responsible for increased resistance in these strains, we complemented the *mexR* E118* and *nalD* T158P evolved strains with wild-type copies of *mexR* or *nalD* in *trans*. As controls, we also complemented *mexR* and *nalD* MPAO1 transposon mutants. In both the evolved strains and the *nalD* and *mexR* MPAO1 transposon mutants, expression of wild-type genes restored aztreonam susceptibility, confirming that functional loss of *mexR* or *nalD* was responsible for the aztreonam resistance phenotype in these strains (Fig. S1). While inactivating mutations in *mexR*, such as the nonsense mutation carried by our strain, are known to increase efflux pump expression (42), the effect of the *nalD* missense mutant was less clear. We therefore measured *mexA* expression in the parental MPAO1 strain and the *nalD* T158P variant using reverse transcription-quantitative PCR (qRT-PCR) analysis and found that the *nalD* mutant had 4-fold-increased *mexA* expression relative to the wild type (unpaired Student’s two-tailed *t* test, *P* = 0.007), consistent with upregulation of efflux system expression in the *nalD* mutant.

10.1128/mBio.00517-17.1 FIG S1  MIC results from *mexR* and *nalD* complementation. Aztreonam susceptibilities of evolved strains and transposon mutants. Strain names and corresponding plasmids are indicated above each image. Download FIG S1, PDF file, 0.4 MB. Copyright © 2017 Jorth et al. 2017 Jorth et al. This content is distributed under the terms of the Creative Commons Attribution 4.0 International license.

**FIG 3:**
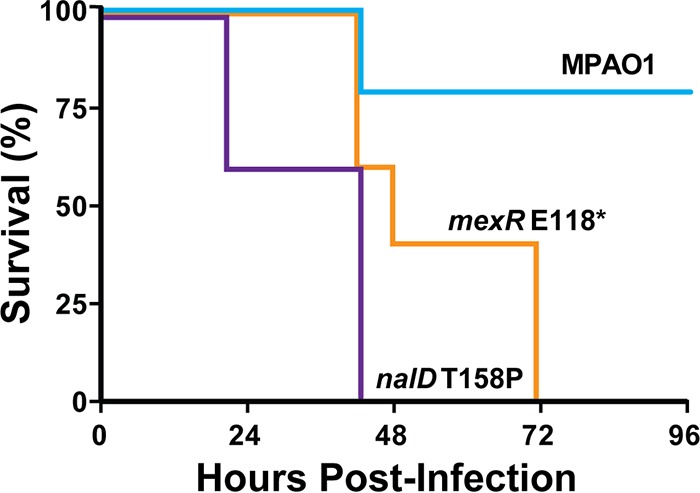
Evolved aztreonam-resistant MPAO1 strains are hypervirulent. Survival of mice subjected to acute pulmonary infections with wild-type PAO1 or mutants with single nonsynonymous mutations obtained through cyclic aztreonam selection (*nalD* T158P and *mexR* E118*). Results are aggregated for *n* = 5 mice per group. Both evolved strains were more lethal than the wild type (*P* < 0.05, log rank test).

The evolved strains were independently used to infect mice in an acute pneumonia model (6). Compared to the wild-type MPAO1 ancestor, both mutant strains caused significantly greater mortality (Fig. 3): hazard ratios indicated that the *mexR* E118* mutant was approximately 8 times more likely to kill mice than the wild-type parent, while infection with *nalD* T158P was nearly 13 times more likely to be lethal. We conclude that selection for aztreonam resistance can also result in hypervirulence phenotypes.

We considered the possibility that secondary mutations could be responsible for the hypervirulence phenotype. Recent work has shown that pf1 phage expression in *P. aeruginosa* infections can influence virulence (44), and we noted that both evolved strains carried mutations in PA0724, a hypothetical pf1 phage coat protein. To verify that these mutations did not affect phage production, we measured phage production in both strains in comparison to the wild-type parent strain (44) but found that neither of the evolved isolates produced more phage particles than the parental strain (Fig. S2), indicating that alterations in phage production are not responsible for the hypervirulence phenotype.

10.1128/mBio.00517-17.2 FIG S2  Phage production in evolved MPAO1 strains. PFU of phage particles purified from biofilm-grown strains was quantified by infecting an MPAO1 strain lacking the pf1 phage. Download FIG S2, PDF file, 0.1 MB. Copyright © 2017 Jorth et al. 2017 Jorth et al. This content is distributed under the terms of the Creative Commons Attribution 4.0 International license.

In order to investigate whether *mexAB-oprM* expression could generally influence virulence across *P. aeruginosa* strains, we similarly characterized an engineered Δ*nalD* mutant in the PA14 lineage. Using qRT-PCR analysis, we confirmed that the *nalD* deletion resulted in an ~4-fold (3.8- ± 0.7-fold) increase in *mexA* expression relative to wild-type PA14, with a concurrent 4-fold increase in aztreonam resistance. As before, mice were infected with PA14 and the PA14 Δ*nalD* mutant (Fig. S3). Statistically significant differences in lethality between the two strains were not observed, although hazard ratios indicated that infection with PA14 Δ*nalD* was 1.5 times more likely to kill mice than infection with the wild type. These results suggest that mutations changing *mexAB-oprM* expression can influence virulence in *P. aeruginosa*, although the strain background clearly influences the effect.

10.1128/mBio.00517-17.3 FIG S3  Increased virulence in PA14 strains. Survival of mice subjected to acute pulmonary infections with wild-type PA14 or *ΔnalD* PA14. Results are aggregated for *n* = 6 mice per group. Though *ΔnalD* results are suggestive of increased virulence for the mutant, the outcome was not statistically significant with the number of mice examined (*P* = 0.5, log rank test). Download FIG S3, PDF file, 0.1 MB. Copyright © 2017 Jorth et al. 2017 Jorth et al. This content is distributed under the terms of the Creative Commons Attribution 4.0 International license.

### Virulence-enhancing aztreonam resistance mutations are found in clinical isolates.

Although CF patients are typically each infected with a single, persistent *P. aeruginosa* strain, lineages diversify over decades of chronic infection and generate clonally related siblings within a patient that are marked by phenotypic differences (27). To evaluate whether virulence-enhancing *nalD* and *mexR* mutations, and other recurrent resistance mutations identified from our evolution experiments, can evolve in naturally occurring CF clinical isolates, we collected 15 clonally related *P. aeruginosa* isolate pairs from CF sputum samples collected from adult patients which differed in aztreonam susceptibility (Table 2). The average MICs were 6.4 ± 6.6 µg/ml and 195.9 ± 100.2 µg/ml for the sensitive and resistant isolate pairs, respectively. Eleven of the resistant isolates exceeded the limits of detection for the assay (≥256 µg/ml).

**TABLE 2:** Aztreonam MICs and mutated resistance genes in clinical *P. aeruginosa* strains

Patient identifier	Aztreonam MIC (μg/ml) for isolate		Mutation(s) in gene^a^:												
	Susceptible isolate	Increased- resistance isolate	*mexR*	*ftsI*	*clpA*	*ampC*	*phoQ*	*mpl*	*nalC*	*nalD*	*dacB*	*PA3206*	*pgi*	*atpD*	*pepA*
3	6	256		G531D, A513T		Q155R, V239A, V356I	G301G	M38fs				R274H			
6	5	58	S88fs	G191D											
12	0.5	256		S543G, A60V			H223Y						G469S		G214S
13	0.19	256		R504C, G63D										H169R	W414*
15	16	256													
17	12	32		A244T											
18	5	24	V16fs												
22	24	256	V5fs	I524S		N347T		P62fs							R26fs
28	8	256		R504H, A482V, N242S		G183S, V239A, V356I		M38fs		L22P		G208D			
31	2	256		Y367C		R126C, G183S									
34	0.19	8													
44	8	256		I524T, T267A	G105E	V239A, D245G			E10E		Y264C				
46	8	256										C272R			
90	0.38	256		A244T	G105R										
Vx611	0.125	256		P527S	E635G			M38fs							

^a^ Fractions of strains with nonsynonymous mutations in each gene were as follows: *mexR*, 0.20; *ftsI*, 0.73; *clpA*, 0.20; *ampC*, 0.33; *phoQ*, 0.13; *mpl*, 0.27; *nalC*, 0.07; *nalD*, 0.07; *dacB*, 0.07; *PA3206*, 0.20; *pgi*, 0.07; *atpD*, 0.07; *pepA*, 0.20.

We used whole-genome sequencing to determine whether recurrently mutated genes from our *in vitro* evolution experiments also occurred in isolates from CF patient lungs (Table 2). In total, 13 of 15 resistant isolates carried one or more nonsynonymous mutations in recurrently mutated genes identified from our evolution experiments. As in our *in vitro* studies, *ftsI* mutations were most frequently identified (11 of 15 resistant isolates), and interestingly, multiple missense *ftsI* mutations occurred in individual resistant clinical isolates. The second most frequently identified mutations affected the *ampC* beta-lactamase gene (5 of 15 resistant isolates), with four of the five affected strains similarly having multiple missense mutations in that gene. Three frameshift mutations in *mexR* and a nonconservative substitution in *nalD* were encountered in four clinical strains, making mutations in *mexAB-oprM* regulators the next most frequent recurrent change. Taken together, these data suggest that *in vitro* selection for aztreonam resistance favorably models mutations arising in *P. aeruginosa* strains that infect CF patients.

## DISCUSSION

In its capacity as an inhaled maintenance therapy, aztreonam has only recently found wide and sustained use in CF patients (23). As such, the potential for clinical *P. aeruginosa* strains to evolve resistance to that agent and the chromosomal mechanisms underlying resistance are incompletely explored. Here, we used *in vitro* experimental evolution and whole-genome sequencing to shed light on these questions and to investigate the biological consequences of chromosomal mutations associated with aztreonam resistance.

We employed two different experimental evolution paradigms, continuous antibiotic selection in liquid medium and cycled antibiotic selection on solid medium, to more comprehensively catalog the mutations arising under aztreonam selection and to explore commonalities and differences between differing resistance selection methods. Notably, higher levels of aztreonam resistance were obtained under continuous aztreonam selection per number of passages. It is possible that cycled isolates with the highest levels of resistance were outcompeted by aztreonam-susceptible siblings that were more fit during periods of drug-free growth, ultimately limiting recovery of lineages with mutations that compromised fitness in the absence of selection. Nevertheless, some recurrently mutated genes were identified across both selection schemes.

Genes that were recurrently mutated as a consequence of *in vitro* aztreonam selection affected multiple pathways (Table 1), and most were found to exhibit variation when clonally related sensitive and resistant *P. aeruginosa* isolates from CF patients were compared (Table 2). Furthermore, high-level resistance was likely a consequence of multiple gene mutations acting in combination. Mutations in four genes were consistently recovered across all parental strains and passaging experiments. The temporally earliest and most frequently occurring of these (*mexR*, *nalC*, and *nalD*) disrupted repression of *mexAB-oprM* (36), which has specificity for aztreonam (40). Surprisingly, 35% of MPAO1 isolates from cyclic passaging had mutations in core components of *mexEF-oprN*, a system which does not efflux aztreonam (40), and all continuously selected MPAO1 strains similarly inactivated *mexEF-oprN* through phase shifts in the positive transcriptional regulator *mexT* (32, 33). The consistency of these mutations and their absence from passaged control strains suggest that loss of *mexEF-oprN* is beneficial during aztreonam selection, possibly by reducing metabolic burdens from unnecessary efflux pump activity (45). Missense mutations in *ftsI*, the target of aztreonam (35, 46), were also frequent (30 of 100 total strains), suggesting that altered target binding is a frequent mechanism underlying resistance (47). Mutations in *ftsI* were also the most commonly identified alteration in the 15 paired clinical samples, with many of the specific mutations clustered around the aztreonam binding site (46). Although the majority of these *ftsI* mutations have not previously been reported, R504H was recently identified in an evolved meropenem-resistant isolate (48), suggesting the potential for cross-resistance to evolve between aztreonam and meropenem.

Some recurrently mutated genes were recovered primarily or exclusively in isolates with high resistance levels, suggesting that they arise as terminal mutations during selection. Twenty-five of 30 continuously passaged isolates carried mutations in *phoQ*, part of a two-component histidine kinase system with multiple regulatory functions which has been implicated in cationic antimicrobial resistance but not previously in aztreonam resistance (49, 50). A second, putative two-component system protein identified in our study, PA3206, was previously linked with aztreonam resistance through an unknown mechanism (51). Intracellular proteases encoded by *clpA* and *clpS* are broadly involved in regulating gene networks (52) and may consequently govern genes associated with aztreonam resistance (52). Variants in the *pepA* protease, involved in alginate production and mucoid colony phenotypes (53), suggest a possible role of exopolysaccharides in aztreonam resistance. We also identified mutations in *aroB*, which encodes 3-dehydroquinate synthase and which has been implicated in resistance to several agents (54, 55), although the mechanism of resistance has not yet been elucidated. Disruptions of *pgi* (phosphoglucoisomerase), *atpA*, and *atpD* (ATP synthase) were observed, all of which are involved in energy metabolism and likely contribute to the slow-growth phenotypes. Penicillin binding protein 4, DacB, has minimal affinity for aztreonam (35) but was also recurrently mutated in terminally evolved isolates and in a single clinical isolate, suggesting an indirect resistance mechanism. Disruption of *dacB* activates beta-lactam resistance, both by activating the *creBC* pathway and by upregulating *ampC* beta-lactamase production through the buildup of peptidoglycan precursors (56, 57); both mechanisms may have activity with aztreonam. Recurrent mutations in *mpl*, also involved in peptidoglycan metabolism, may affect a similar mechanism since mutation of this gene can lead to increased *ampC* expression (58). Recurrent mutations were additionally observed in the coding sequence of *ampC* itself but were found with much higher frequency in clinical isolates than in evolved isolates. Alterations in the substrate binding pocket of *ampC*, including V239A (3/15 clinical isolates), have been reported to increase substrate specificity and can enable resistance to aztreonam and meropenem (28, 48). Last, *orfN* mutations were recovered in evolved PA14 isolates, likely affecting flagellar protein glycosylation (59). Mutations in this gene have been observed in ciprofloxacin-resistant *P. aeruginosa* (60) but have not been reported in aztreonam resistance.

The highest-frequency mutations recovered from both passaging experiments affected expression of the *mexAB-oprM* efflux system (29 of 30 continuously selected isolates and 50 of 70 cycled isolates): mutation of regulatory genes *nalD* and *mexR* in clinical strains is also known to be prevalent (9, 36, 61, 62). Given the multiple roles that this system plays *in vivo*, we undertook further investigation to explore the consequences of these mutations.

First, the *mexAB-oprM* efflux system is an exporter of multiple antibiotics (40). We accordingly found that mutation of *mexAB-oprM* regulators was significantly correlated with ciprofloxacin resistance (40). Somewhat surprisingly, independently of *mexAB-oprM* mutation status, there was marked variability in profiles of resistance to tobramycin and colistin both within and across evolution experiments (Fig. 2), indicating contributions of other mutations selected by aztreonam exposure. Speculatively, it is possible that tobramycin resistance could be mediated by concomitant expression of the *mexXY* system, frequently observed in *mexAB-oprM*-overproducing clinical strains (63), while colistin resistance may reflect the ability of two-component systems to regulate lipopolysaccharide modifications (64). We also noted that resistance to other antibiotics was generally not as high, or was even depressed, for strains subjected to continuous selection, suggesting that the highest-level aztreonam resistance phenotypes are more exclusive for that agent. These findings indicate that selection with aztreonam alone can lead to increased resistance to one or more additional drugs, both dependent on and independent of contributions of the *mexAB-oprM* efflux system.

Second, *mexAB-oprM* has been previously associated with virulence in the host (21). Loss of *mexA* and *oprM* in *P. aeruginosa* through transposon mutagenesis results in reduced virulence in a murine lung infection and reduced fitness relative to wild-type strains during murine gut colonization (21). However, a conflicting study showed that overexpression of *mexAB-oprM* caused attenuation in a nematode infection model (65). Our murine pulmonary infection outcomes for MPAO1 mutant strains suggest that frequently occurring mutations selected by aztreonam exposure result in overexpression of *mexAB-oprM* and produce a hypervirulence phenotype (Fig. 3). The mechanisms underlying this phenomenon are not clear and warrant future study. Components of efflux systems in *Vibrio cholerae* and *E. coli* are involved in toxin secretion (66, 67), raising the possibility that *P. aeruginosa mexAB-oprM* plays a similar role in virulence factor secretion. Interestingly, similar mutations in PA14, a lineage with greater virulence than MPAO1 (68), did not have a statistically significant impact on lethality (see Fig. S3 in the supplemental material), suggesting that inherent strain-specific genotypes influence the impact of *mexAB-oprM* overexpression on virulence. However, as most CF patients are infected with *P. aeruginosa* strains phylogenomically resembling PAO1 (30, 31), the enhanced virulence seen for MPAO1 may be more relevant to that patient population.

Given that high levels of aztreonam resistance arose readily during *in vitro* selection, it is surprising that chromosomally mediated resistance to aztreonam has not become widespread in treated CF patients. Multiple clinical trials have reported the transient recovery of isolates with increased aztreonam resistance during suppressive therapy, remaining far below the drug levels achievable in inhaled therapy (69) and returning to baseline during the off cycle of treatment (25–27): these observations are consistent with a resistance-associated fitness cost. Although we found that aztreonam resistance phenotypes are largely stable in the absence of selection (Table S5), 29 of 30 high-level resistance strains displayed significant reductions in growth rates compared to their wild-type precursors (Fig. 1B). These findings raise the possibility that resistant strains may be overgrown by antibiotic-sensitive lineages in the absence of selection, preventing resistant lineages from coming to dominance. Alternatively, it is possible that mutations promoting aztreonam resistance are otherwise disadvantageous for strains in the complex environment presented by the host lung. Last, this discrepancy may reflect the use of dual antibiotic therapies for management of chronic airway infections. Some CF patients are prescribed alternating treatment cycles of inhaled aztreonam and inhaled tobramycin (70). *In vitro* studies suggest that alternating exposures of aztreonam and tobramycin more effectively kill *P. aeruginosa* biofilms and limit the emergence of resistance than either monotherapy (24), and similar effects may occur *in vivo*.

It is important to acknowledge the limitations of our experimental designs. While we employed two different evolutionary strategies to maximize our ability to detect resistance mutations, no *in vitro* experimental design can perfectly recapitulate conditions present in the CF lung. Both experiments were performed using rich LB medium, and while CF mucus is also nutrient rich, previous work has shown that nutrients available in CF sputum can directly influence bacterial phenotypes (71). Additionally, chronic lung infections in CF often involve *P. aeruginosa* hypermutator strains, mucoid phenotypes, and bacteria living in biofilm-like aggregates (72), and these variables were not represented in our experiments. Despite these considerations, many of the mutations identified in our evolution experiments were also found in clinical isolates with increased aztreonam resistance, suggesting that our experimental approach reasonably approximates *in vivo* processes.

Our findings indicate that a subset of aztreonam resistance mutations may have the unexpected consequence of increasing bacterial virulence. While bacteria in CF infections are conventionally thought to attenuate over time, our previous work isolated hypervirulent strains from patients with late-stage disease (6), suggesting a role for that phenotype during chronic infection. Although the acute murine infection model that we used does not capture the complexity or chronicity of CF lung infections, our results raise the possibility that some strains with aztreonam resistance mutations could have a selective advantage during antibiotic administration and simultaneously increase tissue damage and disease progression. However, there has not been a reported correlation between *P. aeruginosa* aztreonam MIC and patient outcomes, and clinical studies in human subjects will be needed to determine whether an association exists between aztreonam resistance and disease manifestations. In addition, it is possible that mutations selected by exposure to other antibiotics could also increase virulence, such as *nalD* and *mexR* mutations. Future work may identify recurrent resistance-producing mutations that could be targeted to prevent the emergence of resistance or to reduce virulence phenotypes.

## MATERIALS AND METHODS

### Bacterial strains and growth conditions.

PAO1, MPAO1, and all MPAO1 transposon mutants were provided by Colin Manoil (University of Washington) (73). PA14 was obtained from Matthew Parsek (University of Washington), and PA14 transposon mutants were from the transposon library described elsewhere (38). The identity of all MPAO1 transposon mutants was confirmed using insertion-specific PCR before use. The *nalD* deletion mutant was created in strain PA14 using published methods (74). All strains were maintained at 37°C in Luria-Bertani (LB) broth unless otherwise specified.

### Experimental evolution to select for aztreonam resistance.

Continuous aztreonam passaging was performed using 96-well plates, with one replicate passaged per plate to limit the risk of cross-contamination. LB-aztreonam medium spanning eight concentrations in increments of 2-fold serial dilutions was prepared freshly each day and distributed to individual wells, and 5 µl of aerobically grown overnight cultures was inoculated into each well. After overnight aerobic incubation in aztreonam-containing medium, cells from the highest concentration of aztreonam which supported growth were isolated. As before, 5 µl of this culture was reinoculated into aliquots of fresh, aztreonam-containing medium, and the remainder was cryopreserved.

Ten replicates of each continuous-selection strain were selected for increased aztreonam resistance, and a control of each strain was passaged in parallel in the absence of aztreonam. Passaged lineages were examined at two points, (i) when they first became capable of growth at levels of aztreonam compatible with clinical resistance (32 µg/ml) and (ii) when they were maximally adapted to aztreonam. Replicates were streaked onto LB agar containing the maximum concentration of aztreonam supporting growth of the strain, and after overnight incubation, isolated colonies were inoculated in aztreonam broth. The level of aztreonam resistance demonstrated by individual isolates was confirmed using liquid MIC determination, performed according to CLSI guidelines (75), except that LB broth was used.

For the cycled evolution experiment, a single *P. aeruginosa* MPAO1 colony was grown in LB and divided into 95 replicates in a 96-well plate. The cells were grown shaking at 250 rpm at 37°C for 8 h. From each well, approximately 2 µl of cells (~2 × 10^6^ CFU) was stamped onto multiple LB agar plates using a 96-well pin replicator, with each plate containing increasing concentrations of aztreonam (0 to 512 µg/ml). Plates were incubated for approximately 17 h at 37°C, followed by 24 h at 25°C. Isolated colonies were picked for each replicate from the agar plate with the highest concentration of aztreonam that permitted growth, inoculated into a new 96-well plate with fresh LB broth, and grown for 8 h prior to stamping out on aztreonam plates as before. The process was repeated for a total of nine passages in medium with and without aztreonam. After the ninth passage, colonies from each well in the 96-well plate were streaked on LB agar with 4 µg/ml aztreonam to isolate single colonies. A single colony for each replicate was grown in LB broth and archived at −80°C for future experiments.

### Genome sequencing and analyses.

DNA from strains grown under continuous selection was extracted using the Ultraclean microbial DNA isolation kit (Mo Bio). Sequencing libraries were prepared as described elsewhere (76, 77), and sequencing was performed using an Illumina NextSeq500 with 150-bp paired-end chemistries. Cycled MPAO1 colonies were grown overnight in LB, and DNA was isolated from each culture using the Qiagen DNeasy Blood and Tissue kit. Sequencing libraries were prepared using the Illumina Nextera XT DNA library preparation kit and sequenced using an Illumina MiSeq. Average read depths per isolate are provided in Table S7 in the supplemental material.

Sequence analysis of all samples was performed as described previously (76), with minor modifications. Sequence reads were mapped to reference genome PAO1 (AE004091.2) or PA14 (CP000438.1) using bwa-mem (v0.7.12) (78) and SAMtools (v1.1) (79). The average read depth achieved for each strain is reported in Table S7. SAMtools was used for variant calling of single nucleotide polymorphisms (SNPs) and small insertions and deletions (indels), Pindel was used for detection of large indels and structural rearrangement (80), and cn.MOPS (v1.14.2) (81) was used for identification of copy number differences. After primary variant calling, variants observed in both control strains and those passaged in aztreonam were removed from further consideration. Gene variants identified in two or more independently passaged lineages were considered recurrent and were subjected to further analysis.

### Genome sequencing of pairs of aztreonam-resistant and susceptible clinical isolates.

Patients’ consent was obtained before sputum collection, and the study was approved under University of Washington School of Medicine Institutional Review Board approval 31279. CF sputum samples were collected from adults with a history of *P. aeruginosa* infection. To identify pairs of resistant and susceptible *P. aeruginosa* siblings, sputum was treated with Sputolysin, diluted, and plated onto MacConkey agar. Ninety-six colonies were randomly picked from each individual sputum sample and archived for future analyses. The 96 isolates from each sputum sample were grown in LB broth in 96-well plates and stamped onto multiple LB agar plates containing between 0 and 32 µg/ml aztreonam to identify pairs of colonies from individual sputum samples that were sensitive and resistant to aztreonam. When a pair of sensitive and resistant isolates was obtained, they were subjected to Etest strip aztreonam susceptibility testing to determine MICs. Then, each pair was subjected to randomly amplified polymorphic DNA (RAPD) genotyping (5) to determine if they were clonally related. In total, 15 isolate pairs were identified from 15 different subjects. Each of the *P. aeruginosa* clinical isolates was subjected to genome sequencing and variant analysis as described above. Mutations identified in both the sensitive and the resistant isolate in each pair were omitted from further analysis.

### Antimicrobial susceptibility testing.

Antimicrobial susceptibility testing was performed using a combination of broth dilution assays under standard conditions using LB medium and Etest strips (bioMérieux) applied to LB agar in accordance with the manufacturer’s instructions.

### RNA isolation and qRT-PCR analysis.

RNA was isolated from three biological replicates of wild-type and evolved isolates. Each strain was grown to exponential phase (optical density at 600 nm [OD_600_] of 0.8) in LB broth. RNA was isolated using an RNeasy minikit (Qiagen), and RNA integrity was determined with a high-sensitivity RNA ScreenTape and TapeStation instrument (Agilent). RNA samples were treated with 3 U DNase I (Thermo Scientific) to remove DNA contamination, and reaction mixtures were cleaned up with an RNeasy minikit (Qiagen). To ensure removal of DNA contamination, DNase-treated RNA was subjected to PCR amplification of *P. aeruginosa rplU* using Kapa HiFi HotStart ReadyMix (Kapa Biosystems), with wild-type MPAO1 genomic DNA as a positive-control template. DNase-treated RNA was reverse transcribed with NS_6_ random primers using SuperScript III reverse transcriptase (Invitrogen). For each strain, *mexA* expression was determined relative to *rpoD*, by quantitative PCR (qPCR) of cDNA using Kapa SYBR Fast qPCR master mix (Kapa Biosystems).

### Complementation of *mexR* and *nalD* mutant evolved strains and transposon mutants.

Wild-type *mexR* and *nalD* genes were PCR amplified from MPAO1 genomic DNA. Two fragments of the arabinose-inducible vector pMQ72 were amplified for each insert. The *mexR* and *nalD* genes were each inserted downstream of the P_BAD_ promoter in pMQ72 using Gibson Assembly master mix (NEB) to assemble the two vector fragments with the corresponding *mexR* and *nalD* inserts to generate pMQ72::*mexR* and pMQ72::*nalD*. The two constructs were transformed into electrocompetent *E. coli* Top10 cells, and transformants were selected on LB with 20 µg/ml gentamicin. Assembled plasmid constructs were purified with a Qiagen Miniprep minikit. Inserts were confirmed by PCR. The empty-vector control pMQ72 was electroporated into *P. aeruginosa* MPAO1 PW1776 (*mexR* transposon [Tn] mutant), *P. aeruginosa* MPAO1 PW7066 (*nalD* Tn mutant), and the two strains from the cyclic evolution experiment: *P. aeruginosa* MPAO1-AzEvC10 (*nalD* T158P) and *P. aeruginosa* MPAO1-AzEvB8 (*mexR* E118*). pMQ72::*mexR* was electroporated into *P. aeruginosa* MPAO1 PW1776 (*mexR* Tn mutant) and *P. aeruginosa* MPAO1-AzEvB8 (*mexR* E118*) to complement these two strains. pMQ72::*nalD* was electroporated into *P. aeruginosa* MPAO1 PW7066 (*nalD* Tn mutant) and *P. aeruginosa* MPAO1-AzEvC10 (*nalD* T158P) to complement these two strains. Etest aztreonam susceptibility assays were performed on LB with 20 µg/ml gentamicin with and without 20 mM l-arabinose to determine if complementation restored susceptibility.

### Murine lung infections.

Acute murine lung infections were performed as described previously (6). Experiments were approved by the Institutional Animal Care and Use Committee at the University of Washington School of Medicine. Overnight bacterial cultures were grown to mid-exponential phase and diluted to 1 × 10^8^ CFU/ml. Prior to infection, five to six 8- to 12-week-old C57BL/6 mice (Jackson Laboratories) per bacterial strain were anesthetized by intraperitoneal injection with 20 mg ketamine/kg of body weight and 30 mg xylazine/kg in 0.9% saline. Using a 24-gauge angiocatheter, mice were infected with approximately 1 × 10^7^ CFU of MPAO1 or MPAO1 derivatives, or with 5 × 10^5^ CFU of PA14 or PA14 derivatives, which were passively inoculated into the trachea. After infection, mice were allowed to recover for 30 min on a warm blanket. Mice were evaluated twice per day to assess morbidity, and moribund mice were sacrificed with inhaled CO_2_. Survival curves were analyzed with log rank tests.

### Phage expression assays.

Phage expression was assessed as described elsewhere (82). Briefly, strains were cultured as static biofilms, with supernatants harvested after 5 days and applied to a phage-deficient PAO1 strain (*ΔPA0728*). Plaques were counted after overnight incubation.

### Data availability.

Sequence data generated for this study have been submitted to the NCBI Sequence Read Archive (SRA; http://www.ncbi.nlm.nih.gov/sra) under BioProject no. PRJNA377742.
