# Supplementary material for: Evolved Aztreonam Resistance Is Multifactorial and Can Produce Hypervirulence in Pseudomonas aeruginosa
Source: mBio. 2017 Oct 31;8(5):e00517-17. doi: 10.1128/mBio.00517-17 (PMC5666152; doi:10.1128/mBio.00517-17)
Supplement: TABLE S1 [file mbo005173556st1.pdf]

Table S1: Summary of bacterial strains

Isolate Name	Parent Strain	Selection Conditions	Days of Passaging	Aztreonam MIC
A1	MPAO1	Cycled	9	16
A10	MPAO1	Cycled	9	26
A11	MPAO1	Cycled	9	12
A12	MPAO1	Cycled	9	16
A2	MPAO1	Cycled	9	16
A3	MPAO1	Cycled	9	12
A5	MPAO1	Cycled	9	12
A6	MPAO1	Cycled	9	12
A7	MPAO1	Cycled	9	16
A9	MPAO1	Cycled	9	24
B1	MPAO1	Cycled	9	8
B10	MPAO1	Cycled	9	32
B11	MPAO1	Cycled	9	24
B12	MPAO1	Cycled	9	12
B2	MPAO1	Cycled	9	12
B3	MPAO1	Cycled	9	12
B4	MPAO1	Cycled	9	48
B5	MPAO1	Cycled	9	256
B8	MPAO1	Cycled	9	24
C1	MPAO1	Cycled	9	16
C10	MPAO1	Cycled	9	4
C11	MPAO1	Cycled	9	20
C12	MPAO1	Cycled	9	16
C4	MPAO1	Cycled	9	12
C5	MPAO1	Cycled	9	12
C6	MPAO1	Cycled	9	8
C7	MPAO1	Cycled	9	12
C8	MPAO1	Cycled	9	12
D1	MPAO1	Cycled	9	14
D10	MPAO1	Cycled	9	6
D11	MPAO1	Cycled	9	16
D12	MPAO1	Cycled	9	24
D2	MPAO1	Cycled	9	24
D3	MPAO1	Cycled	9	24
D4	MPAO1	Cycled	9	48
D7	MPAO1	Cycled	9	24
D8	MPAO1	Cycled	9	64
D9	MPAO1	Cycled	9	64
E10	MPAO1	Cycled	9	16
E11	MPAO1	Cycled	9	24
E12	MPAO1	Cycled	9	24
E4	MPAO1	Cycled	9	16
E5	MPAO1	Cycled	9	16
E6	MPAO1	Cycled	9	48
E7	MPAO1	Cycled	9	32
E8	MPAO1	Cycled	9	12
E9	MPAO1	Cycled	9	16
F10	MPAO1	Cycled	9	12
F11	MPAO1	Cycled	9	24
F12	MPAO1	Cycled	9	6
F2	MPAO1	Cycled	9	24
F3	MPAO1	Cycled	9	12
F7	MPAO1	Cycled	9	16
F8	MPAO1	Cycled	9	16
F9	MPAO1	Cycled	9	16
G1	MPAO1	Cycled	9	16
G10	MPAO1	Cycled	9	12
G11	MPAO1	Cycled	9	8
G12	MPAO1	Cycled	9	48
G3	MPAO1	Cycled	9	6
G5	MPAO1	Cycled	9	256
G6	MPAO1	Cycled	9	12
G7	MPAO1	Cycled	9	2
H10	MPAO1	Cycled	9	16
H3	MPAO1	Cycled	9	6
H4	MPAO1	Cycled	9	12
H5	MPAO1	Cycled	9	24
H6	MPAO1	Cycled	9	12
H7	MPAO1	Cycled	9	12
H8	MPAO1	Cycled	9	16
LP0	PAO1	Continuous	6	2
HP0	PAO1	Continuous	25	2
LP1	PAO1	Continuous	4	32
HP1	PAO1	Continuous	13	128
LP2	PAO1	Continuous	7	32
HP2	PAO1	Continuous	19	1024
LP3	PAO1	Continuous	7	32
HP3	PAO1	Continuous	25	256
LP4	PAO1	Continuous	8	32
HP4	PAO1	Continuous	36	512
LP5	PAO1	Continuous	8	32
HP5	PAO1	Continuous	35	1024
LP6	PAO1	Continuous	7	32
HP6	PAO1	Continuous	25	1024
LP7	PAO1	Continuous	8	32
HP7	PAO1	Continuous	32	1024
LP8	PAO1	Continuous	12	32
HP8	PAO1	Continuous	43	512
LP9	PAO1	Continuous	5	32
HP9	PAO1	Continuous	17	128
LP10	PAO1	Continuous	4	32
HP10	PAO1	Continuous	15	128
LM0	MPAO1	Continuous	6	2
HM0	MPAO1	Continuous	24	2
LM1	MPAO1	Continuous	4	32
HM1	MPAO1	Continuous	17	256
LM2	MPAO1	Continuous	7	32
HM2	MPAO1	Continuous	32	1024
LM3	MPAO1	Continuous	4	32
HM3	MPAO1	Continuous	21	128
LM4	MPAO1	Continuous	4	32
HM4	MPAO1	Continuous	24	512
LM5	MPAO1	Continuous	5	32
HM5	MPAO1	Continuous	18	256
LM6	MPAO1	Continuous	5	32
HM6	MPAO1	Continuous	27	1024
LM7	MPAO1	Continuous	8	32
HM7	MPAO1	Continuous	17	128
LM8	MPAO1	Continuous	7	32
HM8	MPAO1	Continuous	30	1024
LM9	MPAO1	Continuous	7	32
HM9	MPAO1	Continuous	20	256
LM10	MPAO1	Continuous	4	32
HM10	MPAO1	Continuous	29	1024
L40	PA14	Continuous	4	2
H40	PA14	Continuous	28	4
L41	PA14	Continuous	4	32
H41	PA14	Continuous	15	1024
L42	PA14	Continuous	4	32
H42	PA14	Continuous	39	1024
L43	PA14	Continuous	4	32
H43	PA14	Continuous	26	1024
L44	PA14	Continuous	3	32
H44	PA14	Continuous	26	1024
L45	PA14	Continuous	4	32
H45	PA14	Continuous	20	256
L46	PA14	Continuous	4	32
H46	PA14	Continuous	25	512
L47	PA14	Continuous	4	32
H47	PA14	Continuous	27	1024
L48	PA14	Continuous	3	32
H48	PA14	Continuous	32	1024
L49	PA14	Continuous	4	32
H49	PA14	Continuous	27	512
L4-10	PA14	Continuous	10	32
H4-10	PA14	Continuous	41	1024
PAO1 Parent Strain	PAO1	N/A	N/A	4
MPAO1 Parent Strain	MPAO1	N/A	N/A	2
PA14 Parent Strain	PA14	N/A	N/A	4
