# Supplementary material for: Evolved Aztreonam Resistance Is Multifactorial and Can Produce Hypervirulence in Pseudomonas aeruginosa
Source: mBio. 2017 Oct 31;8(5):e00517-17. doi: 10.1128/mBio.00517-17 (PMC5666152; doi:10.1128/mBio.00517-17)
Supplement: TABLE S2 [file mbo005173556st2.pdf]

Table S2: Temporal staging of mutations during selection for aztreonam resistance

PAO1 Gene	PA14 gene	Number of replicates at 32 µg/mL aztreonam resistance with mutation			Number of terminally evolved replicates with mutation		
		PAO1	MPAO1	PA14	PAO1	MPAO1	PA14
ftsI	*ftsI*	1	5	0	9	7	8
phoQ	*phoQ*	4	2	0	7	9	8
mexR	*mexR*	5	5	4	8	6	5
aroB	*aroB*	3	0	0	9	4	0
nalD	*PA14_18080*	4	2	2	4	2	3
clpA	*clpA*	1	0	0	4	4	4
pepA	*pepA*	1	0	0	2	0	2
nalC	*PA14_16280*	0	2	2	2	3	4
orfN	*orfN*	0	0	2	0	0	9
mpl	*mpl*	0	0	0	0	1	6
clpS	*clpS*	0	0	0	0	0	4
PA3047 (dacB)	*PA3047 (dacB)*	0	0	0	1	1	2
ampC	*ampC*	0	0	0	0	3	0
atpA	*atpA*	0	0	0	0	0	2
atpD	*atpD*	0	0	0	0	0	2
PA3206	*PA3206*	0	0	0	0	2	0
pgi	*pgi*	0	0	0	1	1	3
