# Supplementary material for: Evolved Aztreonam Resistance Is Multifactorial and Can Produce Hypervirulence in Pseudomonas aeruginosa
Source: mBio. 2017 Oct 31;8(5):e00517-17. doi: 10.1128/mBio.00517-17 (PMC5666152; doi:10.1128/mBio.00517-17)
Supplement: TABLE S3 [file mbo005173556st3.pdf]

Table S3: Transposon Mutant MIC Analysis					
Tn Strain Name	Gene	Parent Strain	Average MIC (µg/mL)	Standard Deviation (µg/mL)	Standard Error of the Mean (µg/mL)
PW7954	*ampC*	MPAO1	4.5	2.2	1.1
PW7950	*ampR*	MPAO1	5	1.7	0.9
PW7951	*ampR*	MPAO1	2.5	0.9	0.4
PW9464	*aroB*	MPAO1	2	0	0
PW10412	*atpD*	MPAO1	4	0	0
PW5390	*clpA*	MPAO1	4	0	0
PW5389	*clpA*	MPAO1	4	0	0
PW5392	*clpS*	MPAO1	4	0	0
PW5391	*clpS*	MPAO1	5.3	1.9	0.9
PW8445	*ftsI*	MPAO1	4	2.4	1.2
PW1776	*mexR*	MPAO1	14	3.5	1.7
PW9404	*mexR*	MPAO1	8	0	0
PW7798	*mpl*	MPAO1	3.5	2.6	1.3
PW7799	*mpl*	MPAO1	3	1	0.5
PW7067	*nalD*	MPAO1	10	3.5	1.7
PW7066	*nalD*	MPAO1	4	0	0
PW6368	PA3206	MPAO1	5	1.7	0.9
PW6369	PA3206	MPAO1	4	0	0
PW6112	PBP4	MPAO1	2.5	0.9	0.4
PW6111	PBP4	MPAO1	16	0	0
PW7462	*pepA*	MPAO1	4	0	0
PW8975	*pgi*	MPAO1	9	4.4	2.2
PW3132	*phoQ*	MPAO1	3.5	0.9	0.4
PW3131	*phoQ*	MPAO1	4.5	2.2	1.1
MPAO1	WT Control	MPAO1	2	0	0
PA3303	Tn Control	MPAO1	2	0	0
15755	*ampR*	PA14	25	23.5	7.8
23685	*clpA*	PA14	11	4	1.3
34203	*clpS*	PA14	13	4	1.3
4704	*mpl*	PA14	7	1.8	0.6
31143	*nalC*	PA14	13	4	1.3
27882	*nalD*	PA14	13	4	1.3
35855	*orfN*	PA14	8	0	0
45610	*pepA*	PA14	7	2	0.7
33095	*phoQ*	PA14	7	1.8	0.6
PA14	WT Control	PA14	5	2	0.7
