# Supplementary material for: Evolved Aztreonam Resistance Is Multifactorial and Can Produce Hypervirulence in Pseudomonas aeruginosa
Source: mBio. 2017 Oct 31;8(5):e00517-17. doi: 10.1128/mBio.00517-17 (PMC5666152; doi:10.1128/mBio.00517-17)
Supplement: TABLE S4 [file mbo005173556st4.pdf]

Table S4: Doubling Time of Highly Aztreonam Resistant Isolates Pre and Post LB Passaging

Parent Strains	Sample	Doubling Time of Replicates Before LB Passaging (min)				Average (min)	Standard Deviation	Standard Error	Doubling Time Compared To Parent Strain (p-value)	Doubling Time of Replicates After Unselected Passaging (min)				Average (min)	Standard Deviation	Standard Error	Doubling Time Compared to Parent Isolate prior to unselected passaging (p-value)
	HP-1	106.97	103.92	107.63		106.17	1.62	0.93	2.12E-04	84.53	88.64	101.19	89.90	91.06	6.17	3.09	8.70E-03
	HP-2	158.25	198.04	206.91		187.73	21.16	12.22	2.48E-03	119.10	135.38	140.31		131.60	9.07	5.23	1.30E-02
	HP-3	104.86	99.02	111.08		104.99	4.92	2.84	6.61E-03	164.64	173.29	171.15	155.07	166.04	7.09	3.54	5.90E-05
	HP-4	222.88	189.38	202.67		204.98	13.77	7.95	2.65E-04	90.37	112.52	139.47	110.90	113.32	17.44	8.72	7.15E-04
PAO1	HP-5	180.51	194.16	185.83		186.83	5.62	3.24	1.65E-05	130.54	181.45	165.04		159.01	21.22	12.25	7.37E-02
	HP-6	152.01	137.26	139.19		142.82	6.55	3.78	2.82E-04	117.48	131.03	146.54		131.69	11.87	6.85	1.55E-01
	HP-7	91.32	91.20	101.19		94.57	4.68	2.70	7.21E-02	97.90	80.23	88.64	94.82	90.40	6.75	3.38	2.36E-01
	HP-8	190.43	141.75	172.85		168.34	20.13	11.62	4.51E-03	98.74	127.42	121.39		115.85	12.35	7.13	1.74E-02
	HP-9	114.95	117.09	134.59		122.21	8.80	5.08	4.64E-03	131.78	131.28	172.42		145.16	19.28	11.13	1.00E-01
	HP-10	135.65	131.53	160.82		142.67	12.95	7.48	3.58E-03	155.41	117.88	117.28	129.32	129.97	15.45	8.92	1.88E-01
	HM-1	202.67	256.72	259.61	187.34	226.58	32.06	16.03	1.92E-03	145.62	146.54	126.26	137.80	139.06	8.13	4.07	8.49E-04
	HM-2	156.47	152.68	167.43		158.86	6.25	3.61	3.37E-04	127.89	145.62	125.57		133.03	8.96	5.17	1.44E-02
	HM-3	214.60	199.75	182.41	198.61	198.84	11.39	5.70	5.80E-05	111.26	106.31	112.71	127.89	114.54	8.06	4.03	1.53E-04
	HM-4	190.95	213.28	169.89	167.43	185.39	18.52	9.26	9.44E-04	125.12	126.03	132.79		127.98	3.42	1.98	3.83E-03
MPAO1	HM-5	201.50	198.61	188.87	210.68	199.91	7.78	3.89	1.16E-05	194.16	222.88	208.78		208.61	11.72	6.77	1.25E-01
	HM6	125.34	119.92	124.67		123.31	2.41	1.39	1.80E-03	99.16	103.76	95.08	86.43	96.11	6.38	3.19	9.71E-04
	HM-7	184.84	169.89	151.34		168.69	13.70	7.91	2.02E-03	84.32	108.81	95.74		96.29	10.01	5.78	1.90E-03
	HM8	160.08	189.90	149.71		166.56	17.04	9.84	4.68E-03	140.60	150.36	134.85		141.94	6.40	3.69	6.41E-02
	HM-9	134.85	145.62	145.01	149.39	143.72	5.38	2.69	1.40E-04	156.11	138.35	151.34	136.18	145.50	8.44	4.22	2.97E-01
	HM-10	149.06	173.72	176.37	148.74	161.98	13.11	6.55	8.54E-04	97.90	82.22	95.34	84.12	89.90	6.82	3.41	1.53E-04
	H4-1	255.77	255.77	201.50		237.68	25.59	14.77	1.11E-03	123.78	128.84	112.34		121.65	6.90	3.98	3.46E-03
	H4-2	198.04	225.78	224.32		216.05	12.75	7.36	1.37E-04	110.02	110.55	129.56		116.71	9.09	5.25	4.27E-04
	H4-3	216.61	199.75	180.51		198.96	14.75	8.52	4.18E-04	239.84	251.14	203.87		231.62	20.16	11.64	6.91E-02
	H4-4	195.80	179.11	219.35		198.09	16.51	9.53	6.64E-04	170.73	157.18	156.47		161.46	6.56	3.79	2.17E-02
PA14	H4-5	200.33	164.25	159.71		174.77	18.17	10.49	2.31E-03	116.50	142.33	177.73		145.52	25.10	14.49	1.26E-01
	H4-6	147.17	131.03	147.48		141.89	7.68	4.43	5.35E-04	79.95	74.29	81.74		78.66	3.17	1.83	2.12E-04
	H4-7	172.00	172.85	213.28		186.04	19.26	11.12	1.84E-03	102.23	107.30	97.35	94.31	100.30	4.93	2.47	3.98E-04
	H4-8	147.48	121.82	132.53		133.94	10.52	6.08	3.05E-03	165.43	175.48	161.95		167.62	5.74	3.31	8.24E-03
	H4-9	160.08	148.11	160.45		156.21	5.73	3.31	7.41E-05	133.81	131.28	144.11		136.40	5.55	3.20	1.23E-02
	H4-10	201.50	237.38	175.93		204.93	25.21	14.55	2.62E-03	158.25	156.82	134.33	173.29	155.67	13.91	6.95	1.93E-02
	PAO1	86.54	84.12	87.74		86.13	1.51	0.87		88.30	86.00	78.86		84.38	4.02	2.32	2.98E-01
Controls	MPAO1	90.96	101.93	92.67		95.19	4.82	2.78		85.05	119.51	90.73	92.42	96.93	13.32	6.66	4.31E-01
	PA14	83.61	87.19	86.54		85.78	1.55	0.90		71.75	83.21	76.34		77.10	4.71	2.72	8.12E-02
