# Supplementary material for: Evolved Aztreonam Resistance Is Multifactorial and Can Produce Hypervirulence in Pseudomonas aeruginosa
Source: mBio. 2017 Oct 31;8(5):e00517-17. doi: 10.1128/mBio.00517-17 (PMC5666152; doi:10.1128/mBio.00517-17)
Supplement: TABLE S5 [file mbo005173556st5.pdf]

Table S5: Aztreonam MICs of Terminally Resistant Strains Passaged in Absence of Aztreonam

Parent Strain	Isolate	Initial MIC	MIC Week 1	MIC Week 2	MIC Week 3	MIC Week 4
PAO1	HP0	2	4	4	4	4
	HP1	128	128	128	128	128
	HP2	1024	1024	1024	512	1024
	HP3	256	256	256	256	256
	HP4	512	512	256	256	256
	HP5	1024	1024	1024	512	1024
	HP6	1024	1024	256	256	256
	HP7	1024	512	512	512	512
	HP8	512	512	512	512	512
	HP9	128	128	128	128	128
	HP10	128	128	128	64	64
MPAO1	HM0	2	4	4	4	4
	HM1	256	256	256	128	256
	HM2	1024	128	128	128	128
	HM3	128	64	64	64	64
	HM4	512	512	256	256	256
	HM5	256	256	256	128	256
	HM6	1024	1024	512	512	512
	HM7	128	32	32	32	32
	HM8	1024	128	128	128	128
	HM9	256	256	256	128	128
PA14	H40	2	4	8	8	8
	H41	1024	512	256	128	64
	H42	1024	512	256	256	256
	H43	1024	1024	512	512	256
	H44	1024	1024	512	512	256
	H45	256	256	256	256	256
	H46	512	512	512	512	512
	H47	1024	1024	1024	1024	512
	H48	1024	512	512	512	512
	H49	512	256	512	256	256
	H410	1024	1024	1024	512	512
