# Supplementary material for: Evolved Aztreonam Resistance Is Multifactorial and Can Produce Hypervirulence in Pseudomonas aeruginosa
Source: mBio. 2017 Oct 31;8(5):e00517-17. doi: 10.1128/mBio.00517-17 (PMC5666152; doi:10.1128/mBio.00517-17)
Supplement: TABLE S6 [file mbo005173556st6.pdf]

PAO1 and MPAO1 variants

#CHROM	POS	H-P25-6	LBP-P6c	H-M32-2	LBP-M2c	H-M17-7	LBP-M7c	H-M30-8	LBP-M8c					
AE004091	471358	0	0	0	0	0	0	0	0	missense_variant	*mexR*	PA0424	c.392T>C	p.Leu131Pro
AE004091	471384	0	0	0	0	1	1	0	0	frameshift_variant	*mexR*	PA0424	c.362delC	p.Pro122fs
AE004091	471580	0	0	1	1	0	0	0	0	missense_variant	*mexR*	PA0424	c.170T>A	p.Leu57Gln
AE004091	471608	1	1	0	0	0	0	0	0	stop_gained	*mexR*	PA0424	c.142G>T	p.Glu48*
AE004091	549358	0	0	0	0	0	0	0	0	inframe_insertion	PA0488	PA0488	c.244_245insGCTGGA	p.Gly82_Trp83insTrpSer
AE004091	1279001	0	0	0	0	1	1	0	0	frameshift_variant	*phoQ*	PA1180	c.641delA	p.His214fs
AE004091	1279140	1	1	1	1	0	0	1	1	missense_variant	*phoQ*	PA1180	c.779T>G	p.Val260Gly
AE004091	1279452	0	0	0	0	0	0	0	0	frameshift_variant	*phoQ*	PA1180	c.1101_1128delCCGCGTGGGCGCCCGGCTG	p.Arg368fs
AE004091	1279452	0	0	0	0	0	0	0	0	frameshift_variant	*phoQ*	PA1180	c.1101_1128delCCGCGTGGGCGCCCGGCTG	p.Arg368fs
AE004091	2962829	0	0	1	1	0	0	0	0	inframe_deletion	*clpA*	PA2620	c.1741_1743delCTG	p.Leu581del
AE004091	2962876	1	1	0	0	0	0	0	0	inframe_insertion	*clpA*	PA2620	c.1696_1697insTGC	p.Leu566dup
AE004091	3410604	0	0	0	0	0	0	0	0	frameshift_variant	PA3047	PA3047	c.343_344delGG	p.Gly115fs
AE004091	3411359	0	0	1	1	0	0	0	0	missense_variant	PA3047	PA3047	c.1096G>A	p.Gly366Ser
AE004091	3534546	1	1	0	0	0	0	0	0	missense_variant	*wbpG*	PA3150	c.536C>T	p.Ala179Val
AE004091	3984240	0	0	0	0	1	1	0	0	frameshift_variant	*arnD*	PA3555	c.237_240dupCGCC	p.Trp81fs
AE004091	4006653	0	0	0	0	0	0	1	1	stop_gained	*nalD*	PA3574	c.144C>A	p.Tyr48*
AE004091	4593478	0	0	0	0	0	0	1	1	missense_variant	*ampR*	PA4109	c.403G>T	p.Asp135Tyr
AE004091	4594489	0	0	0	0	0	0	1	1	missense_variant	*ampC*	PA4110	c.461A>G	p.Asp154Gly
AE004091	4594744	0	0	0	0	0	0	0	0	disruptive_inframe_deletion	*ampC*	PA4110	c.720_725delTCCCGG	p.Pro241_Gly242del
AE004091	4949090	0	0	0	0	0	0	0	0	missense_variant	*mraY*	PA4415	c.683T>A	p.Leu228Gln
AE004091	4952765	1	1	0	0	0	0	0	0	missense_variant	*ftsI*	PA4418	c.1579C>T	p.Pro527Ser
AE004091	4953607	0	0	1	1	0	0	0	0	missense_variant	*ftsI*	PA4418	c.737C>T	p.Ala246Val
AE004091	4953614	0	0	0	0	0	0	0	0	missense_variant	*ftsI*	PA4418	c.730G>T	p.Ala244Ser
AE004091	4954458	0	0	0	0	0	0	1	1	missense_variant	*ftsL*	PA4419	c.176G>A	p.Gly59Asp
AE004091	5674842	1	0	0	0	0	0	0	0	missense_variant	*aroB*	PA5038	c.293T>A	p.Ile98Asn
AE004091	6006561	0	0	0	0	0	0	1	1	inframe_deletion	*spoT*	PA5338	c.340_351delGCCCAGGCCGAG	p.Ala114_Glu117del
AE004091	6006561	0	0	0	0	0	0	1	1	inframe_deletion	*spoT*	PA5338	c.340_351delGCCCAGGCCGAG	p.Ala114_Glu117del
AE004091	6006573	0	0	0	0	0	0	1	0	missense_variant	*spoT*	PA5338	c.343C>A	p.Gln115Lys

PA14 variants

#CHROM	POS	H-415-1	LBP-41c	H-439-2	LBP-42c	H-426-3	LBP-43c	H-426-4	LBP-44c					
CP000438	486296	0	0	1	1	0	0	0	0	missense_variant	*mexR*	PA14_05520	c.205A>G	p.Thr69Ala
CP000438	486305	0	0	0	0	0	0	1	1	missense_variant	*mexR*	PA14_05520	c.196G>A	p.Ala66Thr
CP000438	486308	0	0	0	0	1	1	0	0	frameshift_variant	*mexR*	PA14_05520	c.185_192delGCCGCGAC	p.Cys62fs
CP000438	933983	0	1	0	0	0	0	0	0	missense_variant	*ampR*	PA14_10800	c.11C>T	p.Pro4Leu
CP000438	934376	1	1	0	0	0	0	0	0	missense_variant	*ampR*	PA14_10800	c.404A>C	p.Asp135Ala
CP000438	1027736	0	0	0	0	1	0	0	0	disruptive_inframe_deletion	*mpl*	PA14_11845	c.1340_1351delCGGCGCTGGCCG	p.Ala447_Ala450del
CP000438	1027736	0	0	0	0	1	1	0	0	disruptive_inframe_deletion	*mpl*	PA14_11845	c.1340_1351delCGGCGCTGGCCG	p.Ala447_Ala450del
CP000438	1551588	1	1	0	0	0	0	0	0	missense_variant	PA14_18080	PA14_18080	c.32C>A	p.Thr11Asn
CP000438	1651624	0	0	0	0	0	1	0	0	missense_variant	*rhlR*	PA14_19120	c.635T>A	p.Val212Glu
CP000438	2040286	1	1	1	1	1	1	1	1	frameshift_variant	*orfN*	PA14_23460	c.147_148delGG	p.Val50fs
CP000438	2158850	0	0	0	0	1	1	0	0	frameshift_variant	*dacB*	PA14_24690	c.240_241insGC	p.Ala82fs
CP000438	2575762	0	0	0	0	0	0	0	1	inframe_deletion	PA14_29740	PA14_29740	c.1210_1215delCTGGGC	p.Leu404_Gly405del
CP000438	2617146	0	0	0	0	0	0	1	1	frameshift_variant	*clpS*	PA14_30210	c.216delA	p.Ala73fs
CP000438	2617999	0	0	1	1	1	1	0	0	missense_variant	*clpA*	PA14_30230	c.668T>G	p.Val223Gly
CP000438	2618953	1	1	0	0	0	0	0	0	missense_variant	*clpA*	PA14_30230	c.1622C>T	p.Ala541Val
CP000438	2787819	1	1	1	1	1	0	0	0	intragenic_variant	PA14_32015	PA14_32015	n.2787820_2787821insA	
CP000438	3390473	0	1	1	0	1	0	1	1	frameshift_variant&missense_variant	PA14_38000	PA14_38000	c.440_449delGCGGCCGATGinsCGGCCGAT	p.Gly147fs
CP000438	4059802	0	0	0	1	0	0	0	0	missense_variant	*fleN*	PA14_45640	c.457G>C	p.Glu153Gln
CP000438	4085521	0	1	0	0	0	0	0	0	missense_variant	*lasR*	PA14_45960	c.538C>T	p.Arg180Trp
CP000438	4369599	0	0	1	1	0	0	0	0	disruptive_inframe_deletion	*phoQ*	PA14_49170	c.1050_1052delACT	p.Leu351del
CP000438	4369768	0	0	0	0	1	1	0	0	disruptive_inframe_deletion	*phoQ*	PA14_49170	c.881_883delGCG	p.Arg294_Ala295delinsPro
CP000438	4369873	0	0	0	0	0	0	1	1	missense_variant	*phoQ*	PA14_49170	c.779T>G	p.Val260Gly
CP000438	4370337	1	1	0	0	0	0	0	0	stop_gained	*phoQ*	PA14_49170	c.315C>G	p.Tyr105*
CP000438	4371066	0	0	0	0	0	1	0	0	missense_variant	*phoP*	PA14_49180	c.260A>G	p.Lys87Arg
CP000438	4563644	0	1	0	0	0	0	0	0	missense_variant	*mvfR*	PA14_51340	c.808T>G	p.Tyr270Asp
CP000438	4881508	0	0	0	0	0	0	1	1	missense_variant	PA14_54970	PA14_54970	c.1412G>T	p.Gly471Val
CP000438	4979743	0	0	0	0	1	1	0	0	inframe_deletion	*pitA*	PA14_55770	c.1267_1269delGCC	p.Ala423del
CP000438	5116323	0	0	0	0	0	0	1	1	missense_variant	*ftsI*	PA14_57425	c.1579C>T	p.Pro527Ser
CP000438	5116499	0	0	1	1	0	0	0	0	missense_variant	*ftsI*	PA14_57425	c.1403A>T	p.Gln468Leu
CP000438	5117177	0	0	1	1	0	0	0	0	missense_variant	*ftsI*	PA14_57425	c.725A>G	p.Asn242Ser
CP000438	5131710	0	0	1	0	1	0	1	1	intragenic_variant	PA14_57610	PA14_57610	n.5131711_5131744delATGTAGGGCGCAG	
CP000438	5584809	1	1	0	0	0	0	0	0	missense_variant	*panC*	PA14_62590	c.619A>C	p.Thr207Pro
CP000438	5913068	0	1	0	0	0	0	0	0	frameshift_variant	PA14_66320	PA14_66320	c.672_1075del	p.Glu225fs
CP000438	6070062	1	1	1	0	1	1	1	1	intragenic_variant	PA14_68010	PA14_68010	n.6070064delT	
CP000438	6070063	0	1	0	0	0	0	0	0	intragenic_variant	PA14_68010	PA14_68010	n.6070064_6070105delTCCGGGCTGTGGT	
CP000438	6070104	0	0	0	0	1	0	0	0	intragenic_variant	PA14_68020	PA14_68020	n.6070106delA	
CP000438	6070105	0	0	1	0	0	0	0	0	intragenic_variant	PA14_68020	PA14_68020	n.6070105A>C	
CP000438	6521037	0	0	0	0	0	0	1	1	frameshift_variant&stop_lost&splice_regi	*atpC*	PA14_73230	c.348_*29del	p.Ser116fs
CP000438	6521820	0	0	1	1	0	0	0	0	missense_variant	*atpD*	PA14_73240	c.1091A>T	p.Gln364Leu

No Detectable Change
Reversion
New Mutation
