# Supplementary material for: Evolved Aztreonam Resistance Is Multifactorial and Can Produce Hypervirulence in Pseudomonas aeruginosa
Source: mBio. 2017 Oct 31;8(5):e00517-17. doi: 10.1128/mBio.00517-17 (PMC5666152; doi:10.1128/mBio.00517-17)
Supplement: TABLE S7 [file mbo005173556st7.pdf]

Isolate Name	Average Read Depth per site
C-PAO1	100.7118194
HP-0	99.90829056
HP-1	99.9562102
HP-2	100.0347098
HP-3	100.0501274
HP-4	99.97178471
HP-5	100.0171684
HP-6	100.0074294
HP-7	100.0187359
HP-8	99.96375106
HP-9	99.97638706
HP-10	99.9815941
LP-0	99.88590806
LP-1	100.665496
LP-2	99.83650847
LP-3	99.86310254
LP-4	99.79534318
LP-5	99.9469327
LP-6	100.5440315
LP-7	100.0096327
LP-8	100.0259605
LP-9	100.6136826
LP-10	99.9815941
C-MPAO1	100.7123283
HM-0	99.97582659
HM-1	99.74070207
HM-2	99.92654481
HM-3	99.95434889
HM-4	99.90593966
HM-5	100.5890667
HM-6	99.92003661
HM-7	99.89860105
HM-8	100.7413205
HM-9	100.7152532
HM-10	100.7982898
LM-0	100.0167555
LM-1	99.97542927
LM-2	99.89035621
LM-3	100.1345598
LM-4	100.6397143
LM-5	99.99899176
LM-6	99.88336656
LM-7	100.6412096
LM-8	100.696615
LM-9	99.99948838
LM-10	100.5737432
C-PA14	99.92909851
H4-0	99.98259863
H4-1	100.653147
H4-2	100.6867334
H4-3	100.0042436
H4-4	100.006638
H4-5	100.0038648
H4-6	100.0265086
H4-7	100.6673844
H4-8	100.6620444
H4-9	100.6748037
H4-10	100.0317454
L4-0	100.075514
L4-1	100.6316834
L4-2	100.6103274
L4-3	100.5821725
L4-4	100.0010843
L4-5	100.0242101
L4-6	100.0060253
L4-7	100.675567
L4-8	99.95238058
L4-9	100.0074681
L4-10	100.0005091
MPAO1parent	12.79006415
A10	10.71236114
A11	13.9549796
A12	12.32860061
A1	11.16942346
A2	9.970691545
A3	11.30235742
A5	12.57582174
A6	10.18970823
A7	13.03243134
A9	9.724297475
B10	8.87759905
B11	10.43203248
B12	11.4415713
B1	11.2036695
B2	11.17229652
B3	13.24169035
B4	14.3726487
B5	7.620339461
B8	7.991675984
C10	12.77266489
C11	15.38885104
C12	13.03076989
C1	13.97504232
C4	15.80301973
C5	14.79576477
C6	13.36074956
C7	15.08794308
C8	11.86984875
D10	14.2880812
D11	13.47445663
D12	11.37828068
D1	13.40100782
D2	13.36791257
D3	14.24271742
D4	13.27778604
D6	9.878133977
D7	11.39836383
D8	9.465911841
D9	13.1136057
E10	12.55483554
E11	14.32620932
E12	13.70854689
E4	13.26793547
E5	14.7668273
E6	13.47048594
E7	14.33831199
E8	11.48473534
E9	16.37895752
F10	13.14424357
F11	13.684097
F12	11.28737147
F2	14.21120126
F3	13.82713088
F5	14.25149879
F7	14.83550598
F8	12.95767004
F9	18.74456964
G10	13.47554133
G11	14.20009709
G12	14.29412567
G1	14.65655009
G3	14.66041111
G5	16.06639434
G6	14.79765593
G7	12.46047828
H10	11.92111428
H3	8.461040987
H4	14.70598592
H5	15.77130993
H6	12.91715094
H7	10.17531181
H8	14.33134517
P12-A4-S	40.2914041
P12-C9-R	28.11365407
P13-A4-S	57.77514828
P13-G11-R	28.05285068
P15-A12-R	26.54427221
P15-C12-S	36.08543303
P17-G11-S	37.41693112
P17-H3-S	25.17587723
P18-F12-S	45.32652556
P18-F7-R	37.22588565
P22-D10-R	39.89578274
P22-D10-S	55.87124553
P28-G4-S	52.84494519
P28-H1-R	48.54008825
P31-E8-S	37.49464801
P31-F11-R	24.65899406
P34-E8-S	33.98132783
P34-G1-R	39.03609074
P3-A6-R	44.92888677
P3-F5-S	28.55620295
P44-D6-R	53.4681783
P44-D6-S	46.3485251
P46-A10-S	50.66674228
P46-A11-R	40.76540035
P6-A7-S	28.62110761
P6-B8-R	31.71468299
P90-D12-R	33.91229908
P90-H7-S	24.48834893
Vx611-5-B4-R	36.33890151
Vx611-5-D2-S	33.69413403
LBP-41	33.06445682
LBP-42	28.75417994
LBP-43	16.97786941
LBP-44	23.13716997
LBP-M10	63.68990554
LBP-M2	79.95108489
LBP-M7	29.26302694
LBP-M8	41.79604141
LBP-P6	48.53014349
