# Supplementary material for: Evolved Aztreonam Resistance Is Multifactorial and Can Produce Hypervirulence in Pseudomonas aeruginosa
Source: mBio. 2017 Oct 31;8(5):e00517-17. doi: 10.1128/mBio.00517-17 (PMC5666152; doi:10.1128/mBio.00517-17)
Supplement: FIG S1 [file mbo005173556sf1.pdf]

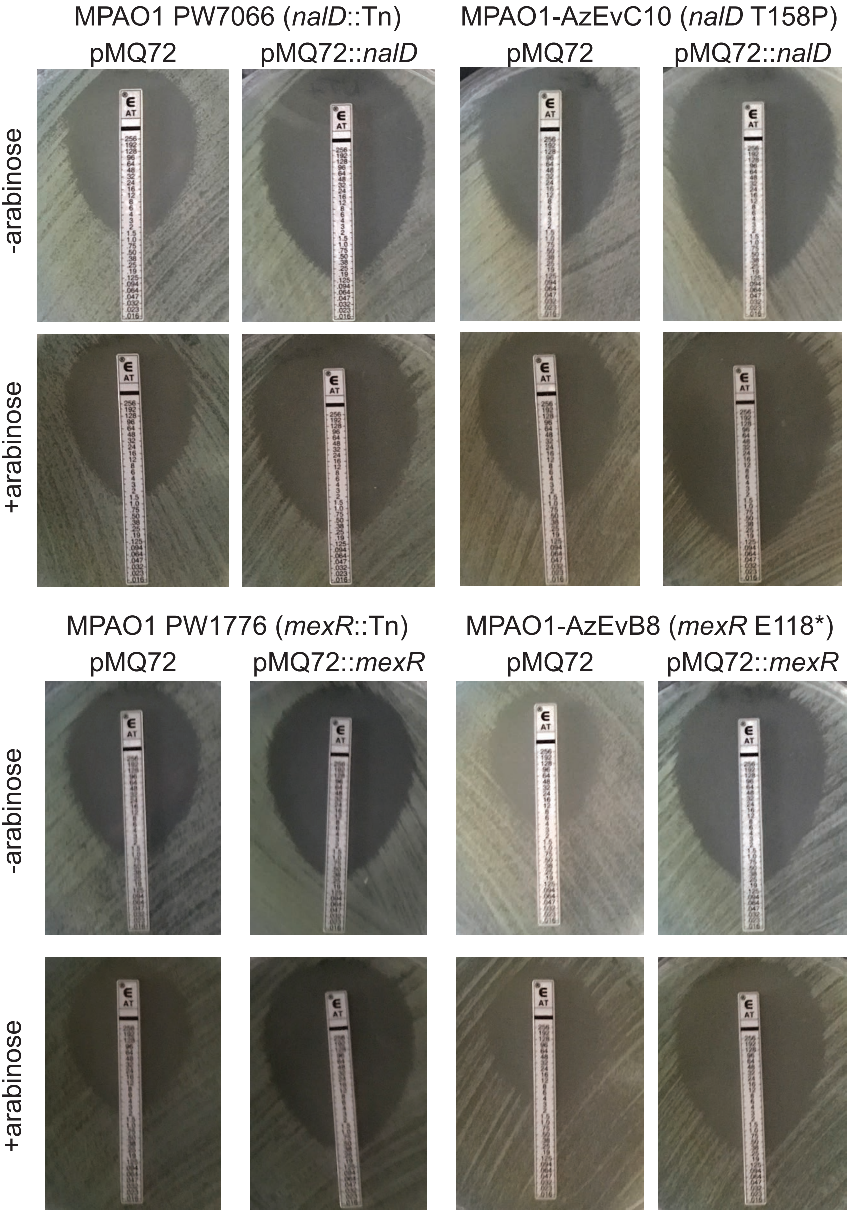
MPAO1 PW7066 (*nalD*::Tn)
MPAO1-AzEvC10 (*nalD* T158P)
pMQ72
pMQ72::*nalD*
pMQ72
pMQ72::*nalD*
-arabinose
+arabinose
MPAO1 PW1776 (*mexR*::Tn)
MPAO1-AzEvB8 (*mexR* E118*)
pMQ72
pMQ72::*mexR*
pMQ72
pMQ72::*mexR*
-arabinose
+arabinose
