# Supplementary figures and images for: Evolved Aztreonam Resistance Is Multifactorial and Can Produce Hypervirulence in Pseudomonas aeruginosa
Source: mBio. 2017 Oct 31;8(5):e00517-17. doi: 10.1128/mBio.00517-17 (PMC5666152; doi:10.1128/mBio.00517-17)

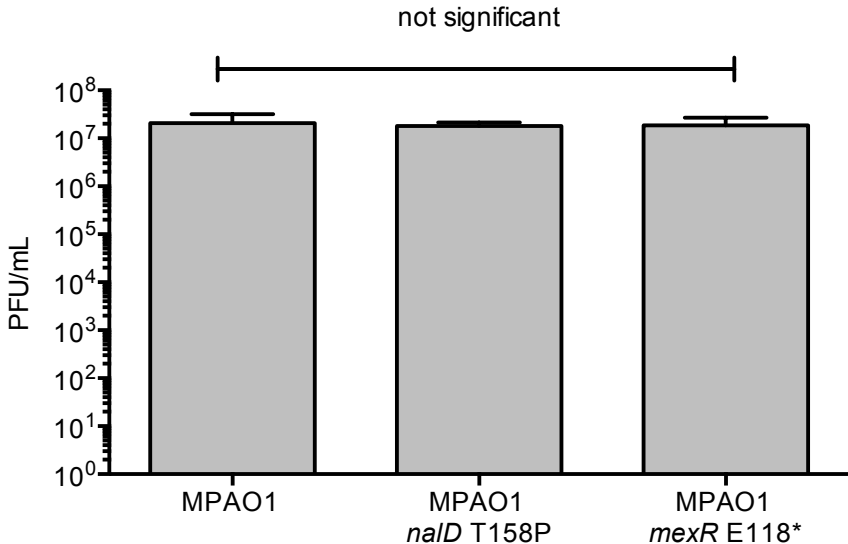

Supplement: FIG S2 [file mbo005173556sf2.pdf]

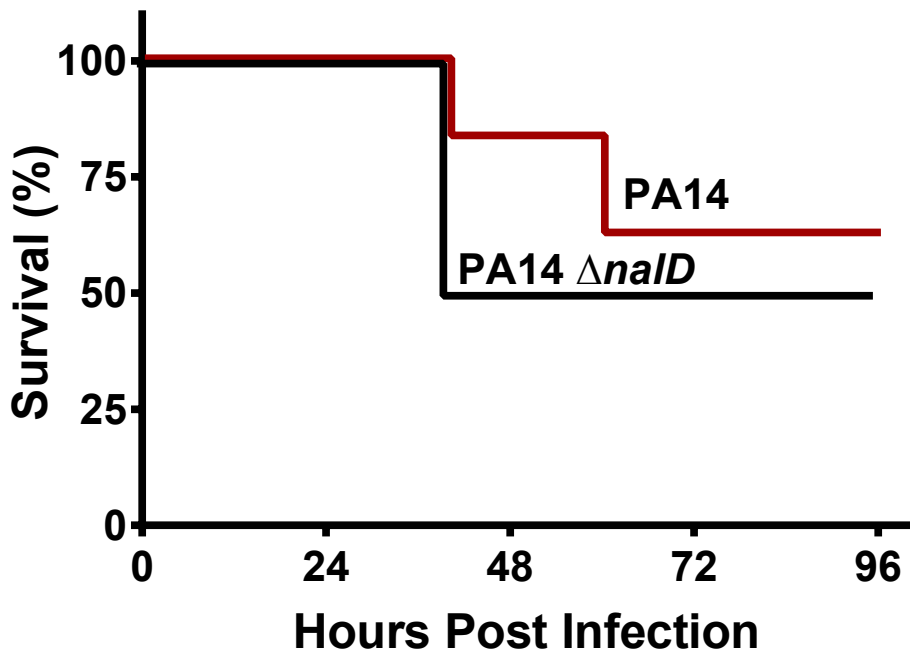

Supplement: FIG S3 [file mbo005173556sf3.pdf]
